# Identification of the Candidate mGlu2 Allosteric Modulator THRX-195518 through In Silico Method and Evaluation of Its Neuroprotective Potential against Glutamate-Induced Neurotoxicity in SH-SY5Y Cell Line

**DOI:** 10.3390/cimb46010051

**Published:** 2024-01-17

**Authors:** Fadime Canbolat, Nigar Kantarci-Carsibasi, Sevim Isik, Suhair Rami Mohammed Shamshir, Münteha Girgin

**Affiliations:** 1 Department of Pharmacy Services, Vocational School of Health Services, Çanakkale Onsekiz Mart University, 17800 Çanakkale, Turkey; 2 Department of Chemical Engineering, Uskudar University, 34662 Istanbul, Turkey; nigar.carsibasi@uskudar.edu.tr (N.K.-C.); munteha.girgin@st.uskudar.edu.tr (M.G.); 3 Stem Cell Research and Application Center (USKOKMER), Department of Molecular Biology and Genetics, Uskudar University, 34662 Istanbul, Turkey; sevim.isik@uskudar.edu.tr; 4 Department of Molecular Biology and Genetics, Uskudar University, 34662 Istanbul, Turkey; suhair.shamsheer.ss@gmail.com

**Keywords:** metabotropic glutamate receptor, cell viability, SH-SY5Y, positive allosteric modulator

## Abstract

Glutamate (Glu) toxicity has been an important research topic in toxicology and neuroscience studies. In vitro and in vivo studies have shown that Group II metabotropic Glu2 (mGlu2) activators have cell viability effects. This study aims to determine a candidate ligand with high mGlu2 allosteric region activity among cytotoxicity-safe molecules using the in silico positioning method and to evaluate its cell viability effect in vitro. We investigated the candidate molecule’s cell viability effect on the SH-SY5Y human neuroblastoma cell line by MTT analysis. In the study, LY 379268 (agonist) and JNJ-46281222 (positive allosteric modulator; PAM) were used as control reference molecules. Drug bank screening yielded THRX-195518 (docking score being −12.4 kcal/mol) as a potential novel drug candidate that has a high docking score and has not been mentioned in the literature so far. The orthosteric agonist LY 379268 exhibited a robust protective effect in our study. Additionally, our findings demonstrate that JNJ-46281222 and THRX-195518, identified as activating the mGlu2 allosteric region through in silico methods, preserve cell viability against Glu toxicity. Therefore, our study not only emphasizes the positive effects of this compound on cell viability against Glu toxicity but also sheds light on the potential of THRX-195518, acting as a mGlu2 PAM, based on in silico absorption, distribution, metabolism, excretion, and toxicity (ADMET) data, as a candidate drug molecule. These findings underscore the potential utility of THRX-195518 against both neurotoxicity and Central Nervous System (CNS) disorders, providing valuable insights.

## 1. Introduction

Glutamate (Glu) is an excitatory neurotransmitter that plays a crucial role in brain functions [1]. Glu interacts with ionotropic Glu (iGlu) and metabotropic Glu (mGlu) receptors, exhibiting pharmacological activity. However, the elevated concentration of Glu in the Central Nervous System (CNS) induces neurotoxic effects [2,3]. The toxic mechanism is initiated by prolonged stimulation of Glu receptors, primarily the N-methyl-D-aspartate (NMDA) receptors (iGlu receptors), due to excessive Glu secretion [1]. Given that Glu toxicity is implicated in neuronal damage across various neurological disorders, it has become a significant focus in toxicology and neuroscience studies [2].

**Figure 1:**
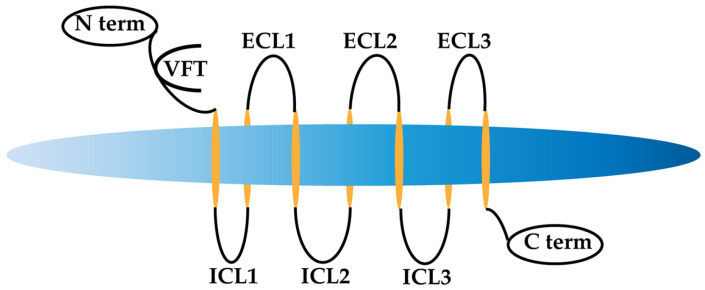
Representation of metabotropic glutamate (mGlu) receptor.

Efforts to prevent and reverse cell damage have predominantly targeted mGlu receptors, particularly the G-protein coupled receptors (GPCRs) in the class C category. Class C mGlu receptors consist of a large extracellular domain (ECL) with a nitrogen (N-terminal) domain, a heptahelical transmembrane (TM) domain, and an intracellular (ICL) domain with a carboxyl-terminal (C-terminal) domain [4]. Notably, these receptors feature a Venus flytrap (VFT) module with an orthosteric region and an ECL containing a cysteine-rich part (Figure 1) [5]. Another distinctive aspect of Class C is the presence of allosteric sites in the TM domain [5]. Allosteric sites render these receptors sensitive to allosteric modulation [4,6].

Class C mGlu receptors encompass eight subtypes (mGlu1–8 receptors). Group I mGlu receptors (mGlu1 and mGlu5) activate phospholipase C through Gq, leading to downstream increases in intracellular diacylglycerol (DAG) and inositol triphosphate (IP3) levels, ultimately resulting in calcium ion (Ca^2+^) release and protein kinase activation [1]. On the other hand, Group II (mGlu2 and mGlu3) and Group III (mGlu4, mGlu7, and mGlu8) receptors inhibit adenylate cyclase (AC) activity via Gi [1]. Group II mGlu receptor agonists, by inhibiting Ca^2+^ flux, are considered potential agents to block Glu-induced cell damage. In vitro and in vivo studies have demonstrated the neuroprotective, antiepileptic, and anxiolytic effects of Group II mGlu agonists [7,8,9,10,11]. Notably, recent research focuses on identifying the mGlu receptor ligands capable of easily crossing the blood–brain barrier (BBB) to enhance neuroprotective efficacy.

Recent advancements in chemoinformatic approaches have shed light on ligand interactions in allosteric domains and orthosteric domains. Allosteric ligand binding may alter receptor conformation and activity, providing subtype-selective modulation, unlike orthosteric agents such as Glu that activate all mGlu receptors [6].

Positive allosteric modulators (PAMs) of mGlu2, including 4-[3-[(2-cyclopentyl-6,7-dimethyl-1-oxo-2,3-dihydroinden-5-yl)oxymethyl]phenyl]benzoic acid (BINA), 1-(cyclopropylmethyl)-2-oxo-4-(4-phenylpiperidin-1-yl)pyridine-3-carbonitrile (JNJ-40068782), and 3-(cyclopropylmethyl)-7-[(4-phenylpiperidin-1-yl)methyl]-8-(trifluoromethyl)-[1,2,4]triazolo [4,3-a]pyridine (JNJ-46281222), have demonstrated reference activity [12,13,14]. Most PAM ligands exhibit a pyrimidine structure, with weights ranging from 250 to 450 g/mol, interacting with the TM and ECL regions of the mGlu2 receptor. In vitro and in vivo studies on the JNJ-46281222 PAM ligand highlight its positive control role, with determined activity values of approximately 8.09 (the negative logarithm of the half maximal effective concentration; pEC50) and an affinity of 8.33 (pKi) [15]. Experimental studies support that mGlu2 activators reduce neuronal cell damage [10,16,17,18].

Three hypotheses shape our study. First, activating the mGlu2 receptor can inhibit cell damage. Second, ligands stimulating the receptor via orthosteric, and allosteric regions may have a cell viability potential against cell damage, with increased selectivity through mGlu2 allosteric site activation. Third, exploring ligands and their interactions with metabotropic receptors is critical in drug discovery. In recent years, an increasing number of three-dimensional (3D) structures for different GPCRs have paved the way for drug development. Considering the impact of mGlu2 receptor activation on human health, finding candidate PAMs through in silico methods and conducting phase studies are crucial steps in drug discovery. Repositioning studies have gained prominence in recent United States Food and Drug Administration (FDA) drug approvals, with approximately one-third of approved drugs resulting from such studies, constituting about 25% of the pharmaceutical industry’s annual revenue [19].

In this context, we aim to identify a candidate molecule with high mGlu2 allosteric activity among cytotoxicity-safe molecules using in silico methods. Subsequently, we evaluate the cell viability activity of this ligand against cell damage formation in vitro. Our study has two phases: systematic in silico simulations for candidate molecule identification and experimental analysis on the SH-SY5Y human neuroblastoma cell line. The initial phase involves virtual biogenic, nutraceutical, and metabolite molecule screening through Zinc15 and Drug Bank libraries. Molecular docking simulations, detailed binding site interaction analysis, absorption, distribution, metabolism, excretion, and toxicity (ADMET) evaluations lead to identifying a potent candidate molecule. This candidate is then validated through in vitro cell culture experiments, comparing its neuroprotective activity with reference molecules (1R,4R,5S,6R)-4-amino-2-oxabicyclo[3.1.0]hexane-4,6-dicarboxylic acid (LY 379268; agonist) and JNJ-46281222 (PAM), both commonly used in the literature for their mGlu2 receptor activation ability. Furthermore, 3-[4,5-dimethylthiazol-2-yl]-2,5 diphenyl tetrazolium bromide (MTT) analysis is employed to investigate the effect of our candidate molecule on cell viability in the SH-SY5Y cell line.

## 2. Materials and Method

### 2.1. In Silico Study

#### 2.1.1. Protein Preparation

In our study, the crystal structures of mGlu2 (PDB id: 7E9G) complexed with small molecules were obtained from the Protein Data Bank and prepared using Schrödinger’s Maestro Molecular Modeling Suite [20,21] and protein preparation wizard module [22]. The retrieved protein structure is first corrected for bond orders and missing hydrogen atoms. All heteroatoms other than the native ligand are removed. However, the water atoms within 5 Å around the binding cleft were kept. If there are any missing side chains or loops, the Prime module was used to fill in these gaps (though this structure did not have any). Protonation states were generated using PROPKA at pH: 7.0. Finally, restrained minimization was carried out using 0.3 Å RMSD and OPLS2005 (Optimized potentials for liquid simulations 2005) force field [23].

#### 2.1.2. Ligand Preparation

Before all docking simulations, the ligands were prepared using the LigPrep module of Maestro, Schrödinger software version 13.4 (New York, NY, USA) [20,21,22]. The ionization states and tautomers were generated using Epik at pH: 7.0 Å ± 2.0 [24]. Stereoisomers were generated using chiralities from the 3D structure of the ligands. Molecules (metabolites, nutraceuticals, and biogenic) delivered from Drug-Bank [25] and Zinc15 [26] databases comprise 2674 and 83,830 molecules, respectively. A total of 86,504 molecules were collected, and a library was prepared. Applying Lipinski’s rule of five (Ro5) [27,28,29], the library was pre-filtered, resulting in 85,716 molecules prepared by LigPrep, generating 121,587 conformers directly docked into the mGlu2 binding site.

#### 2.1.3. Molecular Docking

Molecular docking calculations were conducted using the Glide SP (standard precision) algorithm [30] in the Ligand Docking Module of the Schrödinger Suite. The grid box was generated around the mGlu2 binding cleft centered on the centroid of the co-crystal ligand using the Receptor Grid Generation module. The grid box size was selected to enable docking of the ligands with a length ≤10 Å. The same grid file was used in all docking simulations for a reliable comparison. All the docked ligands were prepared by the LigPrep module before docking, as explained above. Ligands were kept flexible, and Epik state penalties were added to docking scores. To validate the docking protocol, the co-crystallized ligand was docked, and the root-mean-square deviation (RMSD) between co-crystal 1-butyl-3-chloro-4-(4-phenylpiperidin-1-yl) pyridine-2-one (JNJ-40411813) and docked conformation was calculated to be 1.9 Å. Additionally, 85,716 natural source molecules prepared by LigPrep, generating 121,587 conformers, were docked into the mGlu2 binding site. Molecules having high binding affinity (lower than −10 kcal/ mol) were filtered.

#### 2.1.4. Drug Likeness and Absorption, Distribution, Metabolism, Excretion, and Toxicity (ADMET) Analysis

Identified hit and control molecules are subjected to drug-likeness by checking Lipinski’s Ro5 [27,28,29] violations. To predict ADMET, SwissADME (http://www.swissadme.ch (accessed on 28 June 2022)) [31], pkCSM (https://biosig.lab.uq.edu.au/pkcsm/ (accessed on 28 June 2022)) [32,33], and Prediction of Toxicity of Chemicals (ProTox-II; https://tox-new.charite.de/protox_II/index.php?site=home (accessed on 28 June 2022)) [34] servers were used. First of all, the molecular SMILES (Simplified Molecular Input Line Entry System) structures of each molecule were downloaded from the “PubChem” (https://pubchem.ncbi.nlm.nih.gov/ (accessed on 28 June 2022)) page. The downloaded SMILES structures were uploaded to the programs. Each molecule’s physicochemical and ADMET properties were evaluated with the program parameters.

### 2.2. In Vitro Studies

#### 2.2.1. Standard and Reagent

LY 379268 (Cat # 15351) was obtained from Cayman (Ann Arbor, MI, USA), JNJ-46281222 (Cat # GC39393) was obtained from GLPBIO (Montclair, CA, USA), L-glutamic acid (Cat # 56-86-0) was from Sigma Aldrich (St. Louis, MO, USA), Revefenacin Metabolite 1-[[4-[methyl-[2-[4-[(2-phenyl phenyl)carbamoyloxy]piperidin-1-yl]ethyl]carbamoyl]phenyl]methyl]piperidine-4-carboxylic acid (THRX-195518; Cat # 909800-36-8) was obtained from Clearsynth (Mumbai, India), and Dulbecco’s modified Eagle’s medium (DMEM) (Cat # DMEM-HXA) was obtained from CAPRICORN Scientific (Ebsdorfergrund, Germany).

#### 2.2.2. Cell Culture

Human neuroblastoma SH-SY5Y cells were purchased from ATCC (American Type Culture Collection, Manassas, VA, USA), and stored at −196 °C in a liquid nitrogen tank for long-term usage. These cells were routinely cultured in DMEM, supplemented with 15% Fetal Bovine Serum (FBS), 1% L-glutamine, 1% sodium pyruvate, and 100 μg/mL primocin at 37 °C in a humidified atmosphere that contains 5% CO_2_. These cells were thawed and seeded in a 25 cm^2^ cell culture flask, refreshed every 48 h using Complete DMEM, and subcultured every 6 days when their confluency reached 70%.

#### 2.2.3. Preparation of Drugs

LY 379268 (15 mM) was prepared in double distilled water (ddH_2_O). JNJ-46281222 (150.97 mM) and THRX-195518 (150.97 mM) were prepared in dimethyl sulfoxide (DMSO). To prepare L-glutamic acid (569.7 mM, pH 7.4), 5 mg L-glutamic acid was dissolved in 2000 µL DMEM, 1430 µL sodium hydroxide (NaOH; 5 M), and 2535 µL Hydrochloric acid (HCl; 2 M) in a total volume of 5965 µL.

#### 2.2.4. Cell Treatment

To conduct cell treatment and MTT assay, cells were seeded in triplicates at a density of 5500 cells per well in a 96-well plate. L-glutamic acid was employed to induce excitotoxicity in the cells following the study by Palanivel et al. [35]. To analyze the toxic effects of L-glutamic acid, a range of L-glutamic acid doses (0–80 mM) was selected based on doses used in previous studies to assess cell viability. The dose of L-glutamic acid that caused approximately 25% cell death was chosen to establish a mild to moderate excitotoxicity cell model.

Similarly, the cell viability effects of mGlu2 agonist LY 379268, mGlu2 PAM JNJ-46281222, and mGlu2 candidate PAM THRX-195518 at different doses were determined based on those described in previous in vitro studies. In the literature, the protective effect of LY 379268 has been studied at doses of 0.1 µM [36], 2 µM [37], and 100 µM [38]. Therefore, in this study, 0.1, 1, 10, 25, 50, 100, and 150 μM concentrations were applied to the cells to find the optimal safe dose for LY 379268 in cytotoxicity analysis. In the literature, 0.1–1 µM dose ranges of JNJ-46281222 have been studied to determine the protective effect of JNJ-46281222 [14,39]. Our study also investigated a range of 1–1000 nM to determine the optimal safe dose of JNJ-4628122 in cytotoxicity analysis. Since no previous studies on THRX-195518 exist, a broad concentration range (1–150 µM) was employed to establish the optimal safe dosage.

**Figure 2:**
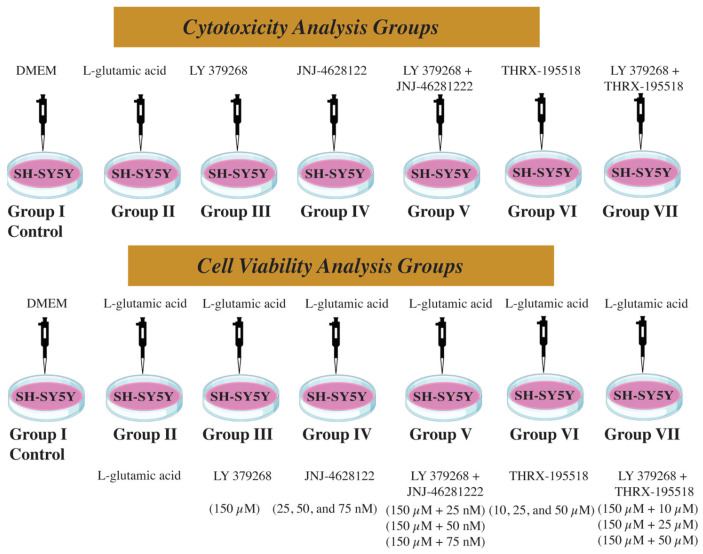
Experimental groups in the study.

The cells were divided into seven groups to determine the optimal safe dose for each molecule. Group I; DMEM control group, Group II; L-glutamic acid group (cells were treated with 20, 40, 60, and 80 mM L-glutamic acid, respectively), Group III; mGlu2 agonist LY 379268 group (cells were treated with 0.1, 1, 10, 25, 50, 100, and 150 µM LY 379268, respectively), Group IV; mGlu2 PAM JNJ-46281222 group (cells were treated with 1, 10, 25, 50, 100, 150, and 1000 nM JNJ-46281222, respectively), Group V; Synergistic effect group for LY 379268 + JNJ-46281222, Group VI; mGlu2 candidate PAM THRX-195518 group (cells were treated with 1, 10, 25, 50, 100, and 150 µM THRX-195518, respectively), Group VII; Synergistic effect group for LY 379268 + THRX-195518 (Figure 2). The optimal safe doses of the molecules used in the cell culture were determined using an MTT cell viability assay.

The MTT assay was used to assess the cell viability effects of each molecule against 25% cell damage caused by glutamic acid after the optimal safe dosages for each molecule were identified by cytotoxicity studies. The experimental groups included in the study are shown in Figure 2.

In the model of 25% cell damage induced by glutamic acid, cells were subjected to pretreatment with the optimal safe doses of LY 379268, JNJ-46281222, and THRX-195518 to investigate the effects of LY 379268, JNJ-46281222, and THRX-195518 on cell viability. Additionally, dual drug combinations (JNJ-46281222 with LY 379268 and THRX-195518 with LY 379268) were applied to the cells (Figure 2). Subsequently, one hour post-pretreatment, the dose of L-glutamic acid causing 25% cell death in cells was uniformly added to all experimental groups. Moreover, a negative control containing DMEM and a positive control containing the dose of L-glutamic acid causing approximately 25% cell death were included. This systematic experiment was designed to investigate the cell viability potential of each agent, both independently and in combination.

#### 2.2.5. 3-[4,5-Dimethylthiazol-2-yl]-2,5 diphenyl tetrazolium bromide (MTT) Assay

Cell proliferation kit, 3-(4,5-Dimethylthiazol-2-yl)-2,5-diphenyltetrazolium bromide MTT, which was purchased from BOSTER (Cat # AR1156) (Pleasanton, CA, USA), was used to measure the viability of the cells 24 h after treatment. The kit converts water-soluble MTT into insoluble formazan, where the formazan will be solubilized, and its concentration will be determined by optical density. In this experiment, 100 µL of culture media (DMEM + 15% FBS) was applied along with 10 µL MTT after removing the supernatant. The plate was incubated for 4 h. After 4 h, a solubilization solution was added to dissolve the formazan crystals, then the plate was incubated again for 16 h. After incubation, the absorbance was measured using an ELISA microplate reader at 570 nm and 100 rpm.

### 2.3. Statistical Analysis

Results were presented as the mean (M) ± standard deviation (SD) of three replicate experiments. All the analysis data were calculated in the GraphPad Prism 9.5.1 version using the ordinary one-way ANOVA multiple comparisons Tukey test.

## 3. Results

### 3.1. In Silico Studies: Library Generation, Virtual Screening, Molecular Docking, and ADMET

**Figure 3:**
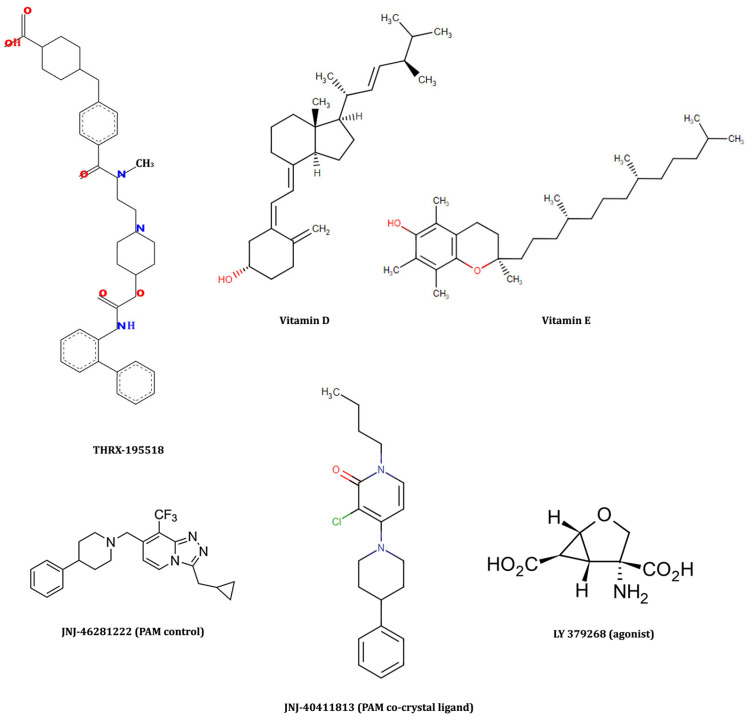
Control drugs and candidate hit molecules retrieved from in silico analysis as mGlu2 activators.

In the current study, we employed a systematic in silico approach to test the protective activity of the molecules (biogenics, metabolites, and nutraceuticals) delivered from the Drug Bank [25] and Zinc15 [26] databases against mGlu2. Our reference control compounds that activate the mGlu2 receptor were LY 379268 (agonist) and JNJ-46281222 (PAM). Out of 121,587 molecules and conformers that were docked to mGlu2 binding sites, three hit molecules (vitamin D, vitamin E, and THRX-195518) were captured as having high docking scores, performing essential interactions with mGlu2 and having acceptable ADMET properties. The chemical structures of these leading compounds and control molecules are depicted in Figure 3.

**Figure 4:**
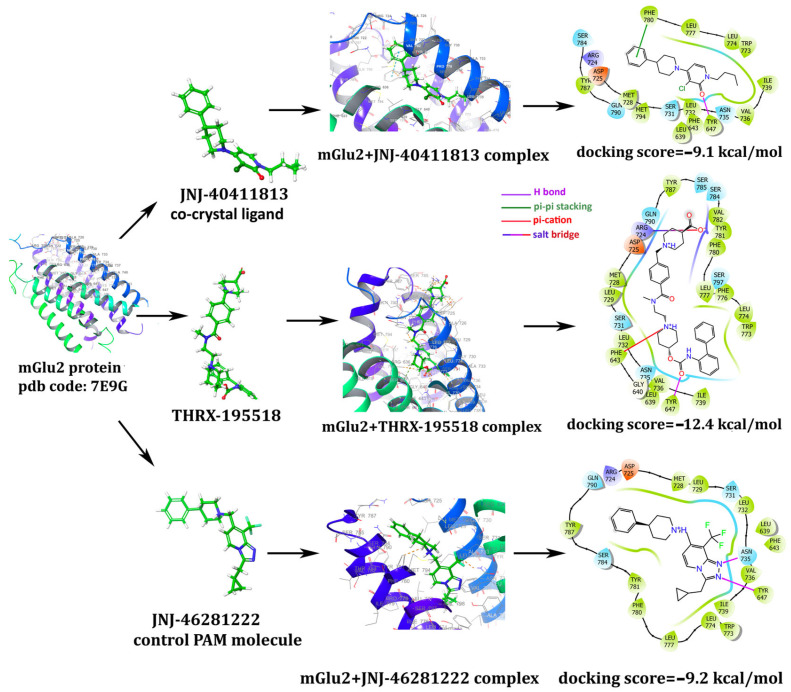
Molecular docking interaction models of mGlu2 (PDB id: 7E9G) in complex with co-crystal ligand JNJ-40411813, control PAM JNJ-46281222 and candidate PAM THRX-195518.

Among the nutraceutical molecules, vitamin D (DB00153; docking scores −9.7 kcal/mol) and vitamin E (DB00163; docking scores −9.3 kcal/mol) stepped forward with high docking scores. Since many literature studies already mention the neuroprotective effects of vitamins D and E, we did not select them as potential novel candidate molecules. Drug bank metabolite molecules screening yielded THRX-195518 (a metabolite of Revefenacin; docking score being −12.4 kcal/mol) as a potential novel drug candidate that has a high docking score and has not been mentioned in the literature so far. Thus, THRX-195518 has been selected among the candidate molecules as the leading compound for which the experimental validations are further continued. THRX-195518 yields higher docking scores than the PAM control (docking score −9.2 kcal/mol) and PAM co-crystal (docking score −9.1 kcal/mol) ligands and maintains crucial binding site interactions, as demonstrated in Figure 4.

JNJ-40411813 (PAM co-crystal ligand) interacts with the mGlu2 binding site with Tyr 647 hydrogen bonding and Phe 780 pi-pi stacking interactions (Figure 4). Our candidate molecule THRX-195518 also makes hydrogen bonding with Tyr 647. It exerts additional interactions, such as a salt bridge through Arg 724 and pi-cation interaction with Phe 643, which boosts the docking scores and paves the way for better binding (Figure 4). Control PAM JNJ-46281222 makes two hydrogen bonds with Tyr 647 and Asn 735 residues (Figure 4). It is worth noting that Tyr 647 is a crucial residue for which hydrogen bonding interaction is present in all investigated cases, i.e., PAM drugs and the candidate molecule THRX-195518.

The SwissADME and pkCSM databases analyze data by considering five distinct evaluation criteria (known as Ro5) to determine the oral absorption potential of molecules [27,28,29,40,41]. The general properties associated with high oral absorption within the program are defined by certain criteria: molecular weight (MW) ≤ 500 g/mol, H-donors ≤ 5, H-acceptors ≤ 10, Rotatable bonds < 10, LogP_Octanol/Water_ (MLogP) ≤ 5, and a topological polar surface area (TPSA) < 140 Å^2^. The results of the physicochemical properties obtained from the SwissADME and pkCSM databases for each molecule are presented in Table 1.

Upon evaluating the MW, H-donor, H-acceptor, TPSA, rotatable bond, and MLogP values of the molecules in Table 1 based on the established program criteria, it was observed that THRX-195518 did not meet the criteria due to its high MW. Although the MW of THRX-195518 exceeds 500, both programs indicated that THRX-195518 possesses drug-like properties. Considering the molecular weights of many FDA-approved drug molecules, the presence of drugs with a molecular weight exceeding 500 has made it understandable why in silico programs assign drug-like properties to THRX-195518. The physicochemical properties of the other molecules conformed to the Ro5 rule, and these molecules were also found to exhibit drug-like properties in both programs.

ADMET properties of the molecules were analyzed in swissADME, pkCSM, and Protox II data tools. When the intestinal absorption (human; % Absorbed) value is <30% in the program output, the intestinal absorption of the molecule is evaluated as low by the pkCSM program [32]. When examining Table 1, it is observed that the molecule with the highest intestinal absorption is JNJ-46281222, while the molecule with the lowest absorption is LY 379268. Considering the lipid solubility levels of molecules, it is noted that both JNJ-46281222 and THRX-195518 exhibit elevated mLogP values, indicating their propensity for lipid dissolution. Crossing the BBB and passing it to the CNS of molecules is evaluated with Log BB and Log PS values, respectively, in the pkCSM web tool. The program shows the distribution of molecules to the brain with a Log BB value of <−1 as low. It is shown by the program that the CNS permeability of molecules with Log PS < −3 is low. When Table 1 is examined, it is observed that JNJ-46281222 can easily pass through the BBB and the CNS. While demonstrating a tendency for lipid solubility, the THRX-195518 molecule’s brain permeability is observed based on its Log PS value; simultaneously, it is speculated that the molecule’s BBB passage is at the threshold. Among the molecules, LY 379268 is observed to have the least ability to penetrate the brain.

Moreover, both JNJ-46281222 and THRX-195518 were determined to function as substrates and inhibitors of cytochrome P450. In the ProTox II web application, the toxicity levels of the molecules are categorized on a scale from 1 to 6, ranging from risky to safe. This toxicity classification is based on the Globally Harmonized System of Classification and Labelling of Chemicals (GHS), which considers the median lethal dose (LD50). Six toxicity classes exist for the ingestion of hazardous substances: Class I (fatal if swallowed), Class II (fatal if swallowed), Class III (toxic if swallowed), Class IV (harmful if swallowed), Class V (may be harmful if swallowed), and Class VI (non-toxic). Among the molecules in the program, THRX-195518 was found to exhibit the least toxic effect (Table 1).

### 3.2. In Vitro Biological Evaluation: Effects of the Molecules on Cell Viability

**Figure 5:**
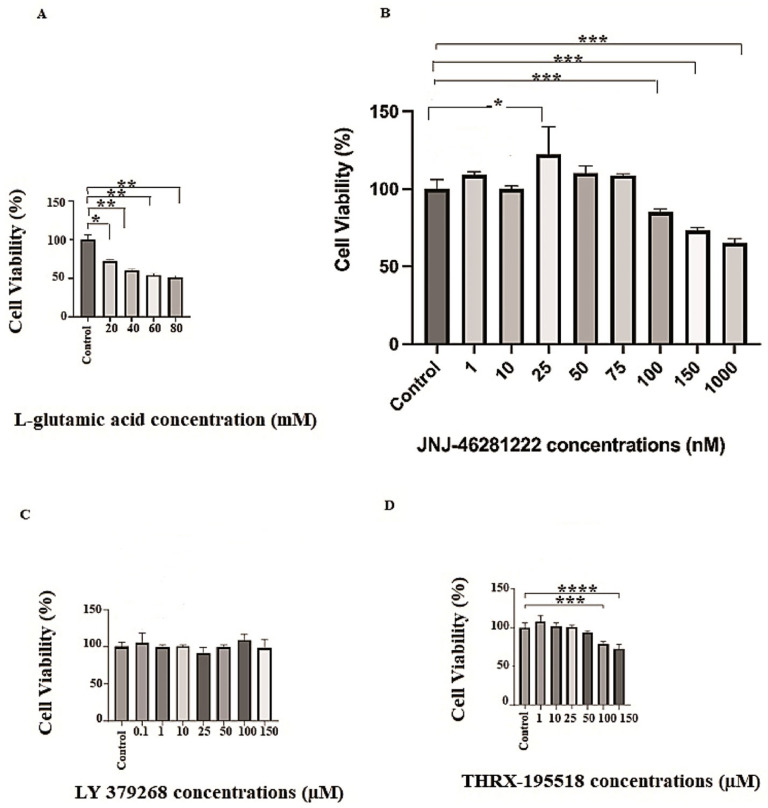
The cytotoxicity analysis of each molecule in different concentrations. (**A**) L-glutamic acid, (**B**) JNJ-46281222, (**C**) LY 379268, (**D**) THRX-195518. Experiments were carried out in triplicate. Data are expressed as mean ± standard deviation (S.D.). Compared to control vs. sample groups ****; *p* < 0.0001,***; *p* < 0.0005, **; *p* < 0.005, *; *p* < 0.05.

This study applied L-glutamic acid to SH-SY5Y cells in the 20–80 mM range to induce cell damage. Figure 5A shows that the cell viability of cells treated with L-glutamic acid significantly decreased compared to the control group and exhibited a cytotoxic effect within the applied dose ranges (*p* < 0.05, *p* < 0.01). To create a mild-to-moderate excitotoxicity model in SH-SY5Y cells, the dose of L-glutamic acid that causes mild-to-moderate death of cells (IC_25_) was chosen as the toxic level in the study. L-glutamic acid resulted in a substantial reduction in SH-SY5Y cell viability at a concentration of 20 mM compared to the untreated control cells (*p* < 0.05) (Figure 5A).

**Figure 6:**
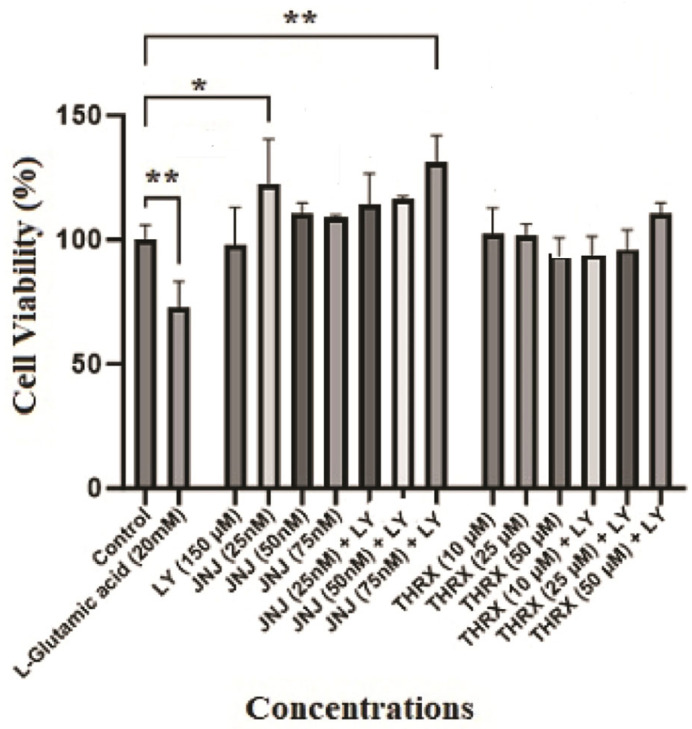
Optimal safe dose analysis results. LY; LY 379268 (150 µM), JNJ; JNJ-46281222, THRX; THRX-195518. Experiments were carried out in triplicate. Data are expressed as mean ± standard deviation (S.D.). Compared to control vs. sample groups **; *p* < 0.005, *; *p* < 0.05.

Different concentrations of the molecules were applied to the cells to determine safe doses for each molecule (LY 379268, JNJ-46281222, and THRX-195518) that do not have a toxic effect on the cells. Different concentrations were applied in the range 1–1000 nM range for JNJ-46281222 (Figure 5B), 0.1–150 µM for LY 379268 (Figure 5C), and the 1–150 µM range for THRX-195518 (Figure 5D). The effects of different concentrations of these three molecules on the viability of cells are shown in Figure 5. The optimal safe dose range for each molecule in cell viability analysis was determined by comparing it with the untreated control cells. Dose ranges that showed similar or higher cell viability than the control group were selected for subsequent analyses. According to these results, the highest concentrations that did not show a significant difference in cell viability compared to untreated control cells were determined as 150 µM for LY 379268, 25, 50, and 75 nM for JNJ-46281222, and 10, 25, and 50 µM for THRX-195518. These doses were determined to be the optimal safe doses for the study (Figure 6). The other higher doses showed significant cytotoxicity relative to control cells (Figure 5); therefore, these were excluded from subsequent analyses.

Concentrations of the molecules (LY 379268; 150 µM, JNJ-46281222; 25, 50, and 75 nM, THRX-195518; 10, 25, and 50 µM) and the combined concentrations of JNJ-46281222 and THRX-195518 with LY 379268 in Figure 6 were determined to be non-toxic. Since the drugs did not show cytotoxicity at the determined doses, the doses indicated in Figure 6 were used to determine the cell viability analysis against Glu-induced cell damage.

**Figure 7:**
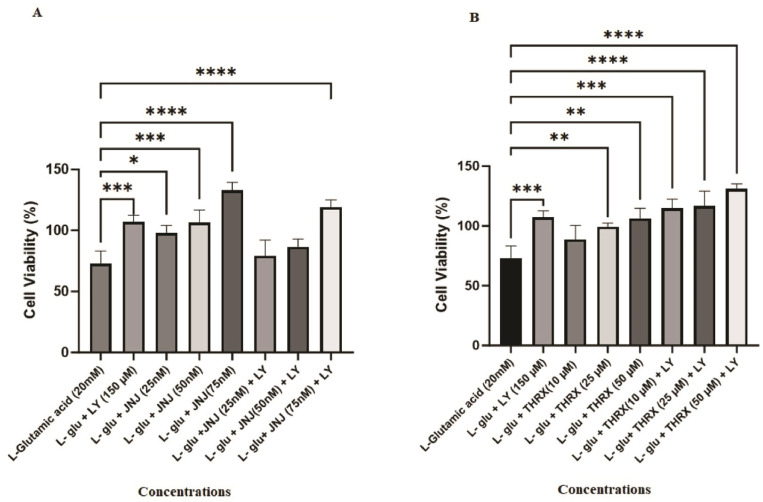
Cell viability activity of molecules against L-glutamic acid toxicity. LY; LY 379268 (150 µM), JNJ; JNJ-46281222, THRX; THRX-195518. (**A**) Single-use and dual-use effect of LY 379268 and JNJ-46281222, (**B**) Single-use and dual-use effect of LY 379268 and THRX-195518. Experiments were carried out in triplicate. Data are expressed as mean ± standard deviation (S.D.). Compared to L-glutamic acid vs. sample groups ****; *p* < 0.0001,***; *p* < 0.0005, **; *p* < 0.005, *; *p* < 0.05.

Figure 7A illustrates that agonist LY 379268 (150 µM) (*p* < 0.001), all concentrations of mGlu2 PAM JNJ-46281222 (25 nM (*p* < 0.05), 50 nm (*p* < 0.001), and 75 nM (*p* < 0.0001)), and the combination of 150 µM LY 379268 with 75 nM JNJ-46281222 as an agonist and PAM dual drug exhibit a significant protective effect on cell viability, reducing cell death induced by 20 mM L-glutamic acid (*p* < 0.001). Additionally, there appears to be a dose-dependent increase in the protective effect of JNJ-46281222 (Figure 7A).

Similarly, cell death induced by 20 mM L-glutamic acid was reduced by agonist LY 379268 (150 µM) (*p* < 0.001), mGlu2 candidate PAM THRX-195518 (25 and 50 µM) (*p* < 0.01), and the combination of 150 µM each of LY 379268 + THRX-195518 in dual-drug agonist and candidate PAM treatment (*p* < 0.001, *p* < 0.0001) (Figure 7B). Figure 7B further demonstrates that the protective effect of THRX-195518 increases dose-dependently.

**Figure 8:**
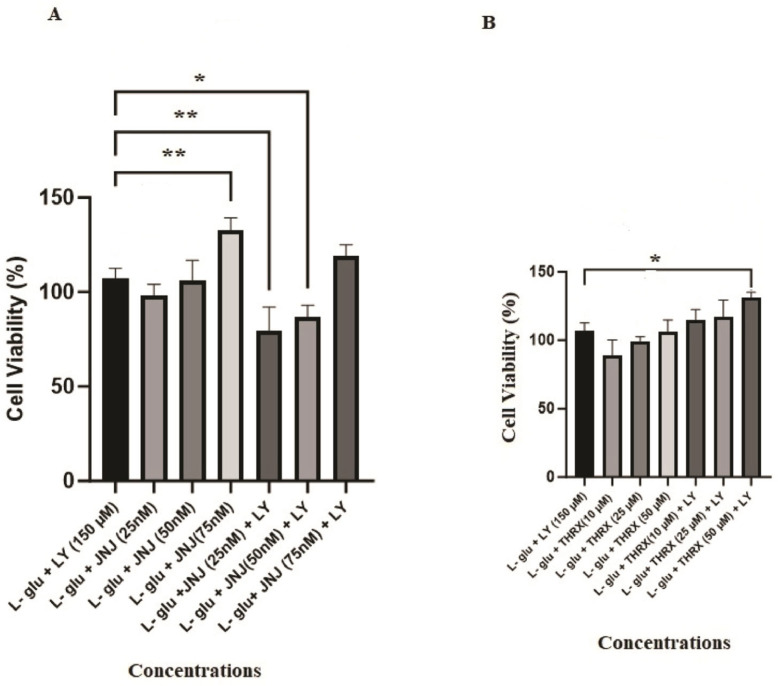
Evaluation of single and dual drug administration to determine the cell viability activity against the activity of LY 379268. LY; LY 379268 (150 µM), JNJ; JNJ-46281222, THRX; THRX-195518. (**A**) Single-use and dual-use effects of LY 379268 and JNJ-46281222, (**B**) Single-use and dual-use effects of LY 379268 and THRX-195518. Experiments were carried out in triplicate. Data are expressed as mean ± standard deviation (S.D.). Compared to LY 379268 vs. sample groups **; *p* < 0.005, *; *p* < 0.05.

As has been seen in Figure 8A, it was found that the application of 75 nM JNJ-46281222 led to a more substantial increase in cell viability compared to 150 µM LY 379268, resulting in a notably higher level of cell viability and exhibiting a pronounced protective effect (*p* < 0.01). However, it was observed that the protective effect of lower doses (25 and 50 nM) of JNJ-46281222, when used in combination with LY 379268 as part of a dual-drug regimen, was inferior to the protective effect observed with the sole use of LY 379268. With an increasing dose, the protective effect of JNJ-46281222 approached that of LY 379268 (Figure 8A).

Figure 8B shows that the protective effect of THRX-195518 at various doses closely resembles that of 150 µM LY 379268. The positive effect of THRX-195518 on cell viability directly correlates with increasing dosage. Notably, the combined use of 50 µM THRX-195518 with LY 379268 in dual drug use demonstrates a synergistic protective efficacy of LY 379268 when used alone. It provides a higher level of cell viability (*p* < 0.05). Furthermore, it is observed that the protective effect increases with increasing dosage (Figure 8B).

**Figure 9:**
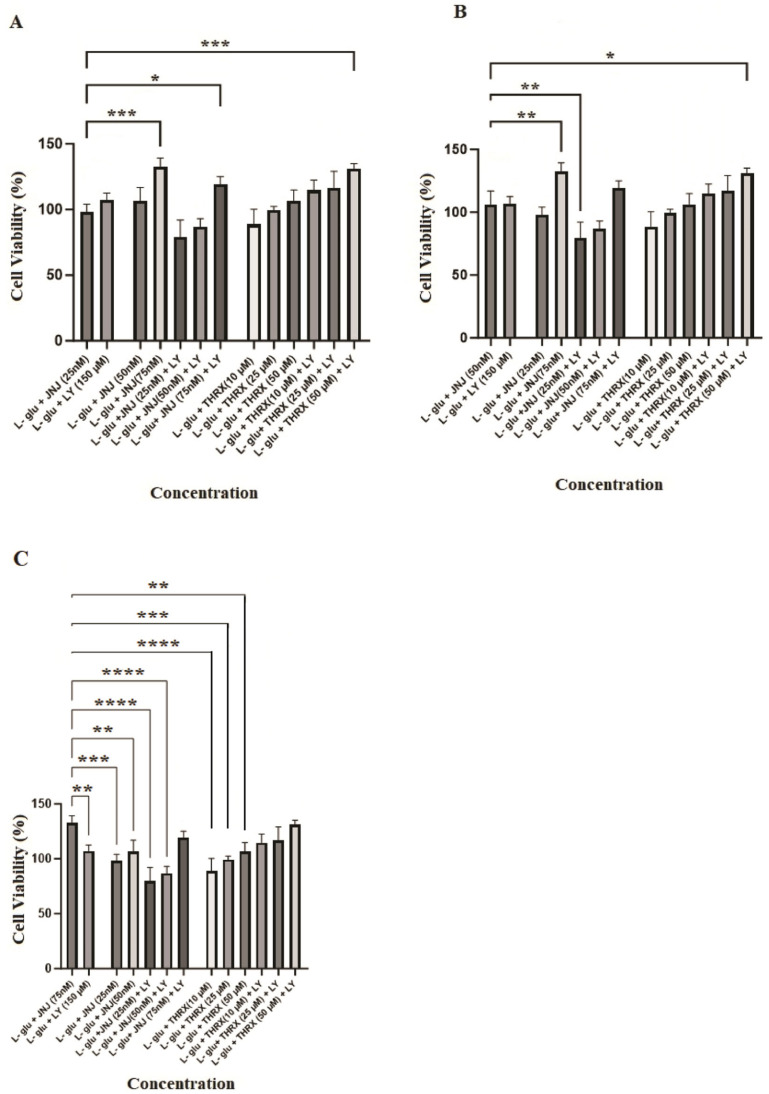
Evaluation of the cell viability activity of different doses of JNJ-46281222 based on single and dual drug administration. LY; LY 379268 (150 µM), JNJ; JNJ-46281222, THRX; THRX-195518. (**A**) Comparison of 25 nM JNJ-46281222 versus other groups, (**B**) Comparison of 50 nM JNJ-46281222 versus other groups, (**C**) Comparison of 75 nM JNJ-46281222 versus other groups. Experiments were carried out in triplicate. Data are expressed as mean ± standard deviation (S.D.). Compared to JNJ-46281222 vs. sample groups ****; *p* < 0.0001,***; *p* < 0.0005, **; *p* < 0.005, *; *p* < 0.05.

Figure 9 shows the concentration-dependent protective effect of JNJ-46281222, which acts as the mGlu2 PAM compared to the other drug groups. It becomes evident that when JNJ-46281222 was applied to the cells at a concentration of 25 nM, the resulting cell viability was significantly lower than that observed with 75 nM JNJ-46281222 (*p* < 0.001), both in its single use and in the dual-drug combinations (150 µM LY 379268 + 75 nM JNJ-46281222 (*p* < 0.05); 50 µM THRX-195518 + 150 µM LY 379268 (*p* < 0.001)). The protective effect of other drug groups closely resembles that of 25 nM JNJ-46281222 (Figure 9A). The cell viability observed following the application of 50 nM of JN-J46281222 to the cells was notably lower than the cell viability observed in both the single use of 75 nM JNJ-46281222 (*p* < 0.01) and in the dual-drug use (50 µM THRX-195518 + 150 µM LY 379268) (*p* < 0.05). However, during dual drug use, it is noteworthy that the protective effect of 150 µM LY 379268 + 25 nM JNJ-46281222 was significantly inferior to that of 50 nM JNJ-46281222. It suggests that achieving a synergistic effect may not be attainable at lower doses. The protective effect of other drug groups closely resembles that of 50 nM JNJ-46281222 (Figure 9B). Moreover, the protective effect of 75 nM JNJ-46281222 was found to be significantly higher than other drug uses. Drug applications exhibiting similar protective effects as 75 nM JNJ-46281222 include 150 µM LY 379268 + 75 nM JNJ-46281222, and THRX-195518 (10, 25 and 50 µM) + 150 µM LY 379268 (Figure 9C).

## 4. Discussion

Drug repurposing (also called drug repositioning) is a growing strategy for identifying new indications for an approved or investigational drug unrelated to the original one. This strategy offers many advantages over developing an entirely new drug and has gained considerable interest in recent research. In the present study, we employed a systematic in silico repurposing strategy, saving time and cost, to obtain powerful protective agents that successfully activate mGlu2. We also tested their cell viability activity by conducting in vitro experiments conducted on neuroblastoma (SH-SY5Y) cell lines. We tested the cell viability activity of the metabolite of Revefenacin, THRX-195518, in comparison to the effects of the mGlu II orthosteric agonist, LY 379268, and mGlu2 PAM, JNJ-46281222 in the human neuroblastoma SH-SY5Y cell line damaged by Glu.

Pe’rez-Benito et al. (2017) investigated the molecular determinants of allosteric modulation of the mGlu2 receptor through in silico and in vitro applications [15]. The study defines it as an “activation switch” rearranged by PAM binding and a “transmission switch” that does not directly participate in ligand interactions but connects the binding site with the outward movement of TM6 for receptor activation and G protein binding. In the study, the “activation switch” for mGlu2 includes the side chains Phe 643, Asn 735, and Trp 773, while the “transmission switch” comprises the amino acids Tyr 647, Leu 738, and Thr 769. Also, the analyzed PAMs have formed a hydrogen bond with Asn 735 in TM5 for allosteric activity [15]. The study includes the PAM molecules, and among them is JNJ-46281222, which we used as the control PAM in our study. In the study, a hydrogen bond has formed between the nitrogen atom of the triazole chain in the molecular structure of JNJ-46281222 and Asn 735. Furthermore, an aromatic interaction has been observed between the triazolopyridine chain of the molecule and Phe 643. In our study, the control PAM JNJ-46281222 has also interacted with the Asn 735 and Tyr 647 regions of the mGlu2 receptor (Figure 4). Our candidate molecule THRX-195518 also makes hydrogen bonding with mGlu2 (PDB id: 7E9G) binding site with Tyr 647. It exerts additional interactions, such as a salt bridge through Arg 724 and pi-cation interaction with Phe 643, which boosts the docking scores and paves the way for better binding (Figure 4). The binding sites of the molecules in our study show similarities with the literature regarding the mGlu2 receptor. THRX-195518 became a potential novel drug candidate with high docking scores (−12.4 kcal/mol) and drug-like properties. THRX-195518 maintains better docking scores, and additional and prolonged binding site interactions as compared to our PAM control and PAM co-crystal ligands (Figure 4). This drug is a metabolite of Revefenacin, which has been previously reported to produce a sustained, long-acting bronchodilator with lower anti-muscarinic-related side effects, i.e., behaved, a competitive antagonist of muscarinic cholinergic receptors. Bourdet et al. (2020) provided insights into the pharmacokinetic and pharmacological properties of Revefenacin and its major metabolite, THRX-195518, in their study. As stated in the study, Revefenacin, when nebulized, demonstrates kinetic selectivity for the M3 muscarinic receptors as a potent and selective antagonist of human M1–M5 muscarinic receptors, exhibiting limited systemic effects and bronchoselective properties. Studies in patients with chronic obstructive pulmonary disease (COPD) also suggested THRX-195518 was a major metabolite because plasma exposure to the metabolite was approximately 4-fold higher than that of Revefenacin. The pharmacological properties of THRX-195518 showed moderate binding affinity for the five human muscarinic acetylcholine receptors. By comparison, Revefenacin exhibited high affinity for all five muscarinic receptor subtypes. THRX-195518 has a tenfold lower binding affinity for the M3 receptor relative to Revefenacin, with receptor occupancy analysis suggesting a minimal contribution of THRX-195518 to systemic pharmacology relative to Revefenacin [42]. Muscarinic receptors belong to the GPCR family. In the literature, THRX-195518 is reported to affect muscarinic receptors moderately. However, the identification of strong binding of THRX-195518 to the mGlu2, another subgroup of the GPCR family, through our docking analysis suggests that THRX-195518 may exert pharmacological effects through the glutamate system. Nevertheless, there is no evidence in the literature specifically investigating the impact of THRX-195518 on the glutamate system or exploring its neuroprotective effects. Therefore, investigating the potential effects that a molecule with high receptor binding affinity, such as THRX-195518, may exert on cell viability becomes crucial.

In the present study, we investigated the neuroprotective effect of THRX-195518 as a mGlu2 activator for the first time. THRX-195518 is a molecule with a piperidine structure and a molecular weight of 598.73 g/mol (Figure 3). The literature reports that most PAM ligands exhibit a pyrimidine structure [42,43]. When examining drug-likeness and ADMET analysis for THRX-195518 and the other two mGlu2 activators in our study (LY 379268 and JNJ-46281222), it is observed that the molecules exhibit drug-likeness (Table 1). We only briefly reference these techniques recently due to the publication of excellent reviews explaining the computational, in vitro, and in vivo methods used to determine drug penetration across the BBB. Predictive models offer advantages such as throughput and the ability to work with virtual structures, and many promising methods have been published. However, a drawback associated with many computational approaches is the limited availability of in vivo data for model construction. Therefore, the pharmacological properties of marketed drugs have been used as a surrogate to imply access to or exclusion from the CNS, an underlying assumption that may not always be correct. Extensive use of in vitro permeability data have been necessary to obtain large datasets; however, these data do not entirely predict in vivo permeability [44]. In our study, when Table 1 was examined, the absorption rate of the molecules was found to be JNJ-46281222 > THRX-195518 > LY 379268. JNJ-46281222 can easily penetrate the BBB, while the passage of THRX-195518 and LY 379268 molecules through the BBB was weaker using in silico methods (Table 1). THRX-195518 generally demonstrates high solubility in lipids. While such a lipophilic characteristic can have significant implications for drug design, bioavailability, and distribution, it alone may not suffice for specific effects, such as neuroprotection. The molecule’s ability to traverse the BBB and its distribution within the CNS are pivotal factors. When the lipophilicity, BBB passage, and CNS distribution of THRX-195518 are collectively evaluated, the analysis results suggest that the accessibility of THRX-195518 to the target region is estimated to be slightly more challenging than that of JNJ-46281222 but easier than LY-379268. Notably, there is a dearth of literature on studies examining the BBB permeability of THRX-195518. Consequently, due to this limitation, the predictive BBB permeation assessments derived from our in silico ADMET data remain unverifiable against existing literature for THRX-195518. In our study, despite the in silico methods showing limited BBB penetration for LY 379268 and THRX-195518, the literature has demonstrated the ability of LY 379268 to cross the BBB through in vitro and in vivo analyses [45,46]. Similarly, supporting the behavior of the candidate PAM THRX-195518 in the CNS through in vitro and in vivo analyses will provide clarity regarding the molecule’s BBB passage property.

Our study’s hypotheses included that mGlu2 activators can protect cell viability during Glu toxicity. The results of the analysis show that mGlu2 activators can positively affect cell viability in SH-SY5Y cells subjected to mild to moderate levels of Glu toxicity. Our analysis results are found to be in support of our hypothesis. In the study, cytotoxicity analysis was conducted for each molecule over a wide concentration range (Figure 5). As a result of cytotoxicity analysis, optimum safe dose ranges were determined for each molecule (Figure 6). The protective effect of molecules in the determined, optimum safe dose ranges was evaluated in SH-SY5Y cell lines through both single and dual drug applications. In our study, the LY 379268 molecule, which binds to the orthosteric region of the mGlu2 receptor, exhibits an agonist effect and has been studied in the literature for its protective effects. It was used as the control agonist. In the study by Durand et al. (2010), the protective effect of the LY 379268 molecule against cell death triggered by nitric oxide was investigated in rat astrocyte cultures [38]. In the study, LY 379268 was used at a concentration of 100 μM. The study provided evidence for the role of LY 379268 in preventing central CNS injury triggered by several inflammatory processes associated with dysregulated nitric oxide production and its protective effect on astrocyte death [38]. In our study, it is observed that 150 µM LY 379268 can significantly preserve cell viability (*p* < 0.001) (Figure 7A). Our analysis results are similar to the literature. Turati et al. (2020) investigated the antioxidant and protective effects of LY 379268 on in vitro-aged astrocytes. The study reported that LY 379268 exhibited antioxidant effects in aged astrocytes and protected hippocampal neurons against neurotoxicity [36]. When examining the literature data and our study results, it is evident that activating the mGlu2 receptor’s orthosteric region can preserve cell viability and provide protective effects.

Treatment with mGlu2 receptor agonists may also have potential limitations regarding tolerance development. mGlu2 PAMs are therefore developed as an alternative, as they increase the endogenous mGlu2 receptor signaling, have greater selectivity than orthosteric agonists, and may maintain activity based on local, transient, and temporal release of Glu, possibly reducing the risk of tolerance [12]. Therefore, in our study, the effects of molecules JNJ-46281222 and THRX-195518, which have shown binding to the allosteric region of the mGlu2 receptor in silico, were analyzed on cell viability activity against Glu toxicity in SH-SY5Y cells. The effects of orthosteric and allosteric binding site activation on cell viability were assessed in conjunction with our study’s data. The JNJ-46281222 molecule, which binds to the allosteric region of the mGlu2 receptor to activate the receptor and has been studied for its protective effects in the literature [12,13,14], was used as the control PAM in our study. Our study investigated the effect of JNJ-46281222 (25, 50, 75 nM) on cell viability in SH-SY5Y cells subjected to toxicity induced by L-glutamic acid. In our study, SH-SY5Y cells subjected to mild to moderate Glu toxicity exhibited preserved cell viability at each dose of JNJ-46281222 (Figure 7A). It was determined that JNJ-46281222 increased cell viability dose-dependently. When the single applications of JNJ-46281222 and LY 379268 were compared, it was observed that, in a dose-dependent manner, JNJ-46281222 increased cell viability more than LY 379268. The effects on cell viability of JNJ-46281222 (25 nM and 50 nM) and 150 μM LY 379268 demonstrate similar significance. However, the highest cell viability is exhibited by 75 nM JNJ-46281222 (*p* < 0.01). Our study observed that 75 nM JNJ-46281222 showed higher cell viability than 150 μM LY379268 (Figure 8A). When the combined use of two molecules that activate different regions of the mGlu2 receptor was examined for its effect on cell viability, it was determined that LY 379268 reduced the protective effect of JNJ-46281222 at different doses and did not result in a significant increase (Figure 8A). Our study observed that the single use of JNJ-46281222 significantly affected cell viability compared to the dual drug application.

In our study, the effect of candidate PAM THRX-195518, which showed high binding to the allosteric region of the mGlu2 receptor in silico docking analysis (Figure 4), on cell viability was investigated at concentrations of 10, 25, and 50 μM (Figure 7B). The cell viability of SH-SY5Y cells subjected to mild to moderate Glu toxicity significantly increased with THRX-195518 at 25 and 50 μM (*p* < 0.01). It was determined that THRX-195518 increased cell viability dose-dependently and exhibited a protective effect. When the protective effects of THRX-195518 and LY 379268 were compared, it was observed that both molecules have similar cell viability (Figure 8B). In our study, the effect of THRX-195518 on cell viability is similar to the effect of 150 μM LY 379268 on cell viability (Figure 8B). When the combined use of two molecules that activate different regions of the mGlu2 receptor was examined for its effect on cell viability, it was determined that the simultaneous use of 50 μM THRX-195518 and 150 μM LY 379268 resulted in higher cell viability compared to the single use of 150 μM LY 379268 (*p* < 0.05). Our study observed that the dual-drug application of THRX-195518 at certain doses significantly increased cell viability and could lead to a synergistic effect (Figure 8B).

In our study, when comparing the cell viability activity of JNJ-46281222 and THRX-195518, it is observed that the effects of the 25 nM and 50 nM JNJ-46281222 doses on cell viability are similar to those of 10, 25, and 50 μM THRX-195518 (Figure 9A,B). The effect of 75 nM JNJ-46281222 on cell viability was higher than that of the single applications of THRX-195518. Moreover, when 50 μM THRX-195518 and 150 μM LY 379268 were used in combination, they showed a higher increase in cell viability compared to the single drug applications of JNJ-46281222 (25 nM and 50 nM). The co-application of THRX-195518 with LY 379268 shows a similar level of cell viability to the single use of 75 nM JNJ-46281222 (Figure 9C).

In the literature, mGlu2 receptors are potential targets for neuroprotective drugs. mGlu2 receptor agonists inhibit Glu release and promote the synthesis and release of neurotrophic factors in astrocytes. The initial studies on the role of mGlu2 receptors in neurodegeneration were conducted in vitro models, including mixed cortical cultures, mesencephalic neuron cultures, and cerebellar granule cell cultures. In these studies, “first-generation” agonists were used. In cortical cultures, these drugs were neuroprotective against neurotoxicity triggered by brief NMDA stimulation, and interestingly, their effects persisted when administered up to one hour after NMDA stimulation. Neuroprotection studies have been extended to “second-generation” mGlu2/3 receptor agonists, such as LY 379268. These drugs have been proven to protect neurons against NMDA toxicity in culture [10]. In addition to the literature, our study provides evidence that activating the orthosteric and allosteric regions of the mGlu2 receptor preserves SH-SY5Y cell viability against Glu toxicity. In our study, the agonist LY 379268, which affects the orthosteric region, demonstrated a strong protective effect at 150 µM. Our findings also establish that JNJ-46281222 and THRX-195518 preserved cell viability by affecting the allosteric region. While the neuroprotective effects of LY 379268 and JNJ-46281222 have been explored in the literature and their therapeutic potential for neurodegenerative diseases investigated, our data are the first to demonstrate that THRX-195518, acting as a mGlu2 PAM, may also be a potential drug molecule with neuroprotective potential against Glu toxicity. Information regarding the pharmacological properties of THRX-195518 in the literature is limited. Therefore, our study sheds light on this molecule’s potential to be used against Glu toxicity and CNS disorders.

A detailed analysis of our results, utilizing in silico analysis methods, demonstrates the high binding affinity of THRX-195518 to the mGlu2 allosteric site. Additionally, it has been observed that the molecule showcases the ability to preserve cell viability in SH-SY5Y cells using the in vitro MTT analysis method. Molecules can manifest their neuroprotective effects through various cellular and molecular mechanisms. Among these mechanisms, molecules can protect nerve cells against harmful agents by reducing neurotoxicity. Furthermore, it is known that molecules can decrease cellular damage and cell death by reducing oxidative stress and exhibiting antiapoptotic effects. Additionally, molecules can regulate cytokine release and control neuroinflammation, protecting nerve cells from inflammation-related damage.

In our study, the protective effect of THRX-195518 against glutamate toxicity in SH-SY5Y cells has been demonstrated in an in vitro setting. However, to fully comprehend the neuroprotective effect of a new candidate molecule, it is crucial to continue experimental studies on the mentioned mechanisms in both in vitro and in vivo environments. Our analysis data highlight, for the first time, the potential neuroprotective effect of THRX-195518, a molecule whose pharmacological effects have yet to be sufficiently discussed in the literature, especially with no prior studies on its neuroprotective effects. These findings are believed to shed light on future studies.

## 5. Conclusions

This study experimentally validates the protective potential of mGlu2 receptor activators using in silico and in vitro models. Generally acknowledged as promising targets for protective drugs, mGlu2 receptors have shown the ability to preserve cell viability in SH-SY5Y cells when activated in orthosteric and allosteric regions. Particularly, the orthosteric agonist LY 379268 exhibited a robust protective effect. Additionally, our findings demonstrate that JNJ-46281222, and THRX-195518, identified as activating the mGlu2 allosteric region through in silico methods, preserve cell viability against Glu toxicity. While the neuroprotective effects of LY 379268 and JNJ-46281222 have been extensively explored in the literature, with their therapeutic potential for neurodegenerative diseases investigated, our study introduces groundbreaking evidence that THRX-195518, functioning as a mGlu2 PAM, may be a candidate drug molecule with protective potential against Glu toxicity. Current literature on the pharmacological properties and drug efficacy of THRX-195518 is limited. Therefore, our study not only emphasizes the positive effects of this compound on cell viability against Glu toxicity, but also sheds light on the potential of THRX-195518, acting as a mGlu2 PAM, based on in silico ADMET data, as a candidate drug molecule. These findings underscore the potential utility of THRX-195518 against both neurotoxicity and CNS disorders, providing valuable insights. However, in addition to our study findings, it is envisaged that further molecular investigations and mechanistic studies in future research may provide deeper insights into the pharmacological efficacy of this candidate molecule when considered as a reference for future studies.

## Figures and Tables

**Table 1:** The physicochemical and absorption, distribution, metabolism, excretion, and toxicity (ADMET) properties of the investigated molecules.

Molecule	Physicochemical Properties								Drug-likeness (Yes/No)
	Molecular Formula	MW g/mol	TPSA Å²	H-Donor	H-Acceptor	Rotatable Bonds	MLOGP (Log P_octanol/water_)	ESOL (Log S)	
LY 379268	C_7_H_9_NO_5_	187.15	109.85	3	4	2	−3.65	1.83	Yes
JNJ-46281222	C_23_H_25_F_3_N_4_	414.47	33.43	0	4	5	4.30	−5.87	Yes
THRX-195518	C_35_H_42_N_4_O_5_	598.73	102.42	2	6	10	3.24	−4.54	Yes
**ADMET**									
**Molecule**	**GI Absorption** **(% Absorbed)**	**BBB Perm.** **(Log BB)**	**CNS Perm.** **(Log PS)**		**P450** **Substrate**	**P450** **Inhibitor**	**Carcinogenicity/** **Ames Mutagenicity** **(Yes/No)**	**Predicted** **LD50 (mg/kg);** **Toxicity Class**	
LY 379268	23.39	−0.56	−3.59		-	-	No/No	50; 2	
JNJ-46281222	91.57	0.55	−1.53		CYP3A4, CYP2D6	CYP1A2, CYP2C19, CYP2C9, CYP3A4	No/No	500; 4	
THRX-195518	62.54	−1.10	−2.47		CYP3A4	CYP2D6	No/No	700; 4	

MW; molecular weight (g/mol), TPSA; topological polar surface area (Å^2^), H-donor: hydrogen donor, H-acceptor; hydrogen acceptor, MLOGP; Moriguchi octanol-water partition coefficient (Log P_octanol/water_), ESOL; estimated water solubility (Log S), GI absorption; gastrointestinal absorption, BBB perm.; blood–brain barrier permeability (Log BB), CNS perm.; central nervous system permeability (Log PS). P450; cytochrome P450 enzyme, CYP2D6; cytochrome P450 2D6, CYP3A4; cytochrome P450 3A4, CYP1A2; cytochrome P450 1A2, CYP2C19; cytochrome P450 2C19, CYP2C9; cytochrome P450 2C9, CYP3A4; cytochrome P450 3A4, LD50; oral acute toxicity- median lethal dose (mg/kg).

## Data Availability

The data obtained in this research are available for consultation through the email address fadime.canbolat@comu.edu.tr. Data are not available on other sites for consultation.
